# Loss of the IR region in conifer plastomes: Changes in the selection pressure and substitution rate of protein‐coding genes

**DOI:** 10.1002/ece3.8499

**Published:** 2022-01-12

**Authors:** Jingyao Ping, Jing Hao, Jinye Li, Yiqing Yang, Yingjuan Su, Ting Wang

**Affiliations:** ^1^ College of Life Sciences South China Agricultural University Guangzhou China; ^2^ College of Life Science and Technology Central South University of Forestry and Technology Changsha China; ^3^ School of Life Sciences Sun Yat‐sen University Guangzhou China; ^4^ Research Institute of Sun Yat‐sen University Shenzhen China

**Keywords:** conifer, gymnosperm, inverted repeat region, rate heterogeneity, selection pressure

## Abstract

Plastid genomes (plastomes) have a quadripartite structure, but some species have drastically reduced or lost inverted repeat (IR) regions. IR regions are important for genome stability and the evolution rate. In the evolutionary process of gymnosperms, the typical IRs of conifers were lost, possibly affecting the evolutionary rate and selection pressure of genomic protein‐coding genes. In this study, we selected 78 gymnosperm species (51 genera, 13 families) for evolutionary analysis. The selection pressure analysis results showed that negative selection effects were detected in all 50 common genes. Among them, six genes in conifers had higher *ω* values than non‐conifers, and 12 genes had lower *ω* values. The evolutionary rate analysis results showed that 9 of 50 common genes differed between conifers and non‐conifers. It is more obvious that in non‐conifers, the rates of *psbA* (*trst*, *trsv*, *ratio*, *dN*, *dS*, and *ω*) were 2.6‐ to 3.1‐fold of conifers. In conifers, *trsv*, *ratio*, *dN*, *dS*, and *ω* of *ycf2* were 1.2‐ to 3.6‐fold of non‐conifers. In addition, the evolution rate of *ycf2* in the IR was significantly reduced. *psbA* is undergoing dynamic change, with an abnormally high evolution rate as a small portion of it enters the IR region. Although conifers have lost the typical IR regions, we detected no change in the substitution rate or selection pressure of most protein‐coding genes due to gene function, plant habitat, or newly acquired IRs.

## Graphical abstract

There are significant differences in the selection pressure of 12 genes and the evolution rate of 9 genes between conifer and non‐conifer. This difference seems to be not related to the loss of typical IR regions but related to habitat. And the newly obtained short IR region plays a role in stabilizing the genome. The ycf2 located in the IR region showed a reduced substitution rate, providing evidence for the IR region a reduced substitution rate. PsbA is undergoing dynamic changes, and part of it enters the IR region, showing an abnormally high substitution rate.

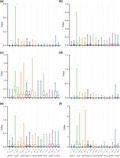

## 1 INTRODUCTION

The plastid genomes (plastomes) of most land plants have a highly conserved quadripartite structure, consisting of a large single‐copy (LSC) region and a small single‐copy (SSC) region separated by a pair of inverted repeat (IR) regions (Wicke et al., 2011). Due to selective pressure on photosynthesis‐related elements, plastids have highly conserved gene content and order (Ruhlman & Jansen, 2014). And due to their highly conservation, a large copy number, lack of recombination, and uniparental inheritance, plastomes have been used to evaluate phylogeographic relationships, phylogeographic histories, and evolutionary events (Barrett et al., 2016; Julian et al., 2017; Moore et al., 2006; Pacheco et al., 2019; Shaw et al., 2014; Wang et al., 2020).

Inverted repeat regions are important in replication initiation (Heinhorst & Cannon, 1993), genomic stability (Maréchal & Brisson, 2010; Palmer & Thompson, 1982), and gene conservation (Palmer & Thompson, 1982; Wolfe et al., 1987). IR changes alter the size of the plastid genome (Kwon et al., 2020). From green algae to angiosperms, IR regions typically contain at least four rRNAs and five tRNAs (Mower & Vickrey, 2018). However, the IR region, as a hot spot of structural rearrangement, often contracts, expands, or is lost. In related species, the boundary of the IR region changes little, resulting in the gain or loss of a small number of genes (Downie & Jansen, 2015; Li et al., 2016; Ping, Li, et al., 2021; Wicke et al., 2014). The IR region of *Pelargonium*, *Psilotum*, and Trochodendraceae has undergone large‐scale expansion, gaining a large number of genes from the SC region (Chumley et al., 2006; Grewe et al., 2013; Sun et al., 2013). The IR region is absent in the plastomes of some plant lineages, such as *prasinophyceans* (Lemieux et al., 2014), *trebouxiophyceans* (Turmel et al., 2015), *streptophytes* (Lemieux et al., 2016), Ulvophyceae (Cai et al., 2017), and Euglenaceae (Karnkowska et al., 2018) among algae; Pinaceae, Cupressophytes (Li, Gao, et al., 2016; Wu, Lin, et al., 2011; Wu, Wang, et al., 2011), and Taxaceae (Zhang et al., 2014) among gymnosperms; and the putranjivoid clade of Malpighiales (Jin et al., 2020), *Tahina spectabilis* (Arecaceae) (Barrett et al., 2016), legumes (Palmer & Thompson, 1981, 1982), *Carnegiea gigantea* (Cactaceae) (Sanderson et al., 2015), and *Erodium* species (Geraniaceae) (Blazier et al., 2016; Guisinger et al., 2011; Ruhlman et al., 2017) among angiosperms.

The sequence substitution rate in the IR region differs from that in the SC region. In some legumes with missing IR regions, the synonymous substitution rate of genes entering the SC region from the IR region is similar to that of those already in the SC region (Perry & Wolfe, 2002). The IR region has a low substitution rate in *Cycas* (Wu & Chaw, 2015). Zhu et al. (2016) found that the synonymous substitution rate of genes in the SC region in vascular plants (angiosperms, gymnosperms, and ferns) is 3.7‐fold that of genes in the IR region; after the transfer of genes between the SC and IR regions, the substitution rate becomes consistent. Some genes in the IR region have high synonymous substitution rates in some species (*Pelargonium*, *Plantago*, and *Silene*). In ferns, *psbA*, *rps7*, 3'‐*rps12*, and *ycf2* showed a reduced substitution rate and increased GC content after entering the IR region (Li, Kuo, et al., 2016). Recently, Ping, Feng, et al. (2021), Ping, Li, et al. (2021) reported that the substitution rate of 3'‐*rps12* in the IR region is lower than that of 5'‐*rps12* in the SC region in ferns and gymnosperms.

The IR region has important effects on genome stability and the evolutionary rate (Maréchal & Brisson, 2010; Palmer & Thompson, 1982; Wolfe et al., 1987). During gymnosperm evolution, conifers lost the IR region. *Araucaria cunninghamii* (Araucariaceae) is native to southeastern coastal areas of Oceania and is the main afforestation tree species in tropical and subtropical regions. It is one of the top five park tree species worldwide and an important garden ornamental plant, and it is cultivated widely in China. *Callitropsis funebris* (Cupressaceae) is endemic to China and distributed widely; it grows rapidly and has a wide range of uses and strong adaptability. Its phylogenetic classification is a focus of research (Zheng & Fu, 1978). As these two species are important representatives of their genera, we sequenced their plastomes. In this study, we selected 78 gymnosperms and 2 Polypodiales (outgroup) and analyzed selection pressure and the evolutionary rate using the maximum likelihood method. We investigated whether the selection pressure and evolutionary rate of protein‐coding genes in conifers differed according to the presence of the IR region; whether the selection pressure and evolutionary rate of genes that enter the IR region differ from those that enter the SC region; and the heterogeneity of gymnosperm evolutionary rates.

## 2 MATERIALS AND METHODS

### 2.1 Sequencing and sequence preparation

Fresh leaves of *Callitropsis funebris* (E113°35’, N23°15’) and *Araucaria cunninghamii* (E113°200’, N23°90’) were taken from the campus of South China Agricultural University. Specimens were stored in Herbarium of the College of Life Sciences, SCAU (specimen no.: PJY‐NYS1910 and PJY‐BM1910). Plastid genomic DNA was extracted using the DNASECure Plant Genomic DNA Extraction Kit (Tiangen) and double‐ended sequencing was performed on Illumina HiSeq2500 platform. Clean reads filtered by Trimmomatic V0.32 (Bolger et al., 2014) were spliced and assembled in Velvet V1.2.03 (Zerbino & Birney, 2008). The genes were predicted by the DOGMA Program (Wyman et al., 2004) and the plastome was mapped using the online site OGDRAW v1.3 (https://chlorobox.mpimp‐golm.mpg.de/OGDraw.html) after gene annotation (Greiner et al., 2019). Finally, the sequence information was submitted on the Banklt Platform (https://www.ncbi.nlm.nih.gov/WebSub/), and Genbank accession no. is MT227812 and MT227813, respectively.

In addition, 76 gymnosperms and 2 Polypodiales (as outgroups) were downloaded from the NCBI database. A total of 78 gymnosperms cover 13 families and 51 genera (Table 1). Genious prime 2020 (Kearse et al., 2012) software was used to extract 50 protein‐coding genes. And performed sequence alignment and correction through the ClustalW (codons) module in MEGA X (Kumar et al., 2018).

**TABLE 1:** The Information of sampled species

Order	Family	Species name	GenBank accession no.	Species name	GenBank accession no.
Cycadales	Cycadaceae	*Cycas revoluta*	NC_020319	*Cycas szechuanensis*	NC_042668
		*Cycas panzhihuaensis*	NC_031413	*Cycas taitungensis*	NC_009618
	Boweniaceae	*Bowenia serrulata*	NC_026036		
	Zamiaceae	*Stangeria eriopus*	NC_026041	*Zamia furfuracea*	NC_026040
		*Ceratozamia hildae*	NC_026037	*Encephalartos lehmannii*	NC_027514
		*Dioon spinulosum*	NC_027512	*Lepidozamia peroffskyana*	NC_027513
		*Macrozamia mountperriensis*	NC_027511		
Ginkgoales	Ginkgoaceae	*Ginkgo biloba*	NC_016986		
Gnetales	Gnetaceae	*Gnetum montanum*	NC_021438	*Gnetum ula*	NC_028734
		*Gnetum parvifolium*	NC_011942	*Gnetum gnemon*	NC_026301
Ephedrales	Ephedraceae	*Ephedra equisetina*	NC_011954	*Ephedra intermedia*	NC_044772
		*Ephedra foeminea*	NC_029347	*Ephedra sinica*	NC_044773
Welwitschiales	Welwitschiaceae	*Welwitschia mirabilis*	EU342371		
Cupressales	Cupressaceae	*Cryptomeria japonica*	NC_010548	*Cupressus tonkinensis*	NC_039562
		*Taiwania cryptomerioides*	NC_016065	*Cupressus gigantea*	NC_028155
		*Taiwania flousiana*	NC_021441	*Cupressus sempervirens*	NC_026296
		*Cunninghamia lanceolata*	NC_021437	*Callitropsis funebris*	MT227813
		*Juniperus monosperma*	NC_024022	*Callitropsis nootkatensis*	KP099642
		*Juniperus recurva*	NC_042763	*Callitropsis vietnamensis*	KX832629
		*Taxodium distichum*	NC_034941	*Hesperocyparis lusitanica*	MH121051
		*Taxodium mucronatum*	NC_045277	*Chamaecyparis formosensis*	NC_034943
		*Calocedrus formosana*	NC_023121	*Chamaecyparis hodginsii*	NC_036996
		*Glyptostrobus pensilis*	NC_031354	*Thuja occidentalis*	NC_042177
		*Metasequoia glyptostroboides*	NC_027423	*Thuja sutchuenensis*	NC_042176
		*Callitris rhomboidea*	NC_034940		
	Taxaceae	*Cephalotaxus sinensis*	MF977938	*Taxus fuana*	NC_038099
		*Cephalotaxus oliveri*	NC_021110	*Pseudotaxus chienii*	NC_041503
		*Amentotaxus argotaenia*	NC_027581	*Torreya fargesii*	NC_029398
		*Amentotaxus formosana*	NC_024945		
	Sciadopityaceae	*Sciadopitys verticillata*	NC_029734		
Pinales	Pinaceae	*Cedrus deodara*	NC_014575	*Picea neoveitchii*	NC_043913
		*Pinus massoniana*	MF564195	*Keteleeria davidiana*	NC_011930
		*Pinus yunnanensis*	NC_043856	*Tsuga chinensis*	NC_030630
		*Pseudolarix amabilis*	NC_030631	*Larix sibirica*	NC_036811
		*Pseudotsuga sinensis*	NC_016064	*Larix decidua*	NC_016058
		*Abies fargesii*	NC_042775	*Abies fanjingshanensis*	NC_042777
Araucariales	Podocarpaceae	*Nageia nagi*	NC_023120	*Retrophyllum piresii*	NC_024827
		*Podocarpus lambertii*	NC_023805	*Dacrydium elatum*	NC_045880
		*Dacrycarpus imbricatus*	NC_034942	*Manoao colensoi*	NC_044893
	Araucariaceae	*Agathis dammara*	NC_023119	*Araucaria heterophylla*	NC_026450
		*Wollemia nobilis*	KP259800	*Araucaria araucana*	NC_045394
		*Araucaria cunninghamii*	MT227812	*Araucaria bidwillii*	NC_045395
		*Araucaria angustifolia*	NC_039155		
Polypodiales	Polypodiaceae	*Lepisorus clathratus*	NC_035739		
	Athyriaceae	*Athyrium anisopterum*	NC_035738		

### 2.2 Analysis of phylogenetic relationships

We constructed the phylogenetic relationships based on the common gene dataset (the two Polypodiales as the outgroup). MEGA X and PAUP4.0 (Swofford, 2002) were used to construct the NJ (neighbor‐joining) tree and MP (maximum‐parsimony) tree, respectively. RaxmlGUI2 software built ML (maximum‐likelihood) tree with GTRGAMMAI substitution model and 1,000 bootstrap (Stamatakis, 2014). BI (Bayesian inference) tree was built with MrBayes 3.2.6 software (Huelsenbeck & Ronquist, 2001). We reconstructed a background tree combined with the published phylogenetic relationship to analyze selection pressure and evolution rate, finally.

### 2.3 Analysis of selection pressure

Codeml program in PAML4.9 was used to analyze the selection pressure (Yang, 2004). The M0 (one‐ration) model assumes that each branch has the same value of *ω* under the Branch model. The Model 2 (two‐ratio) assumes the foreground branch and the background branch have different *ω* values. The likelihood ratio test of M0 and Model 2 can be used to detect the difference of selection pressure between the foreground branch and the background branch.

### 2.4 Analysis of evolutionary rate

We used HyPhy 2.2.4 software (Pond et al., 2005) to calculate the evolution rate, which is based on the maximum likelihood method and in the context of the phylogenetic tree. The transition rate (*trst*), transversion rate (*trsv*), and *trsv*/*trst* (*ratio*) of each branch were calculated under the nucleotide *HKY85* substitution model with the local parameter. Similarly, the synonymous (*dS*) and non‐synonymous (*dN*) substitution rates and *dN*/*dS* (*ω*) of each branch were calculated under the codon *MG94*×*HKY85*×*3_4* substitution model with the *rate Het*. parameter and *4 rate classes*. IBM SPSS v22.0 (IBM Corporation, 2014) software was used to perform the Mann–Whitney U test and Spearman's rank correlations test on related parameters.

## 3 RESULTS

### 3.1 Plastome characteristics

The 76 gymnosperms were divided into two types according to the presence of a typical IR region: 56 gymnosperms lacked a typical IR region (SC‐56; conifer) and 22 gymnosperms had a typical IR region (gnetophytes, cycads, and *Ginkgo biloba*; IR‐22; non‐conifer). For SC‐56 (Appendix S1), the genome size ranged from 117,720 bp (*K*. *davidiana*) to 146,723 bp (*A*.* heterophylla*), with an average of 130,858 bp. The GC content ranged from 34.3% (*T*. *sutchuenensis*) to 39.1% (*C*.* davidiana*), with an average of 36.2%.

**FIGURE 1:**
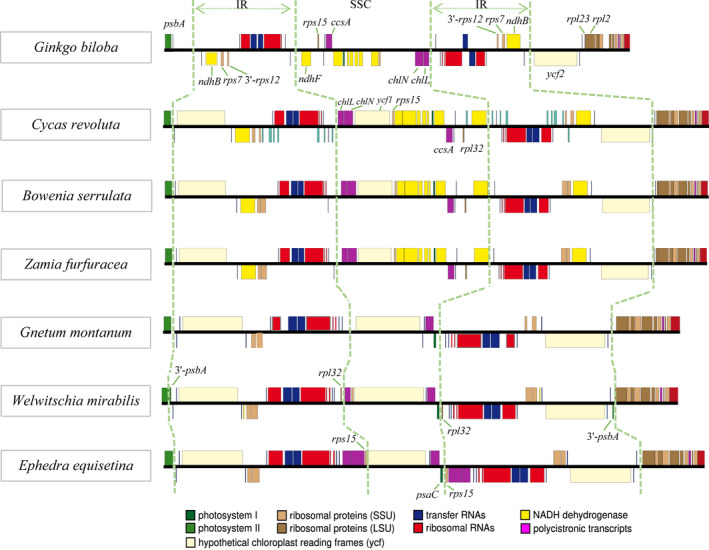
Comparison of IR regions between different taxa. Different color blocks represent different gene types. In species with typical IR regions, there were two protein‐coding genes (*rps7* and 3’‐*rps12*) located in the IR region. In *Ginkgo biloba*, the *ycf2* was located in the LSC region due to contraction of the IR region. Gnetophytes lost *ndh* genes, and in *Welwitschia mirabilis*, 3’‐*psbA* entered IR region

For IR‐22 (Table 2), the genome size ranged from 109,518 bp (*Ephedra equisetina*) to 166,341 bp (*Macrozamia mountperriensis*), with an average of 142,888 bp. The GC content ranged from 36.6% to 40.1%, with an average of 38.7%. The size of the IR region ranged from 17,732 bp (*G*. *biloba*) to 26,137 bp (*Ceratozamia hildae*). Compared with other groups, the plastome of the gnetophytes was the smallest, and the proportion of SSC region was also the smallest (7.2–9.3%), but the proportion of the IR region was largest (16%–18.9%). The three groups all contained the protein‐coding genes *rps7* and 3'‐*rps12* in the IR region, and *ndhB* was present in *G*. *biloba* and cycads. All plants, except *G*. *biloba*, possessed *ycf2*. Individually, 3'‐*psbA* was present in *Welwitschia mirabilis*, *Gnetum parvifolium*, and *Gnetum gnemon*, *rpl32 was* detected in *W*.* mirabilis*, and *rps15* was detected in *E*. *equisetina* (Figure 1).

**TABLE 2:** Plastome structure information of 22 species with typical IR regions

Species name	Genome size/bp	GC/%	LSC/bp	LSC percentage	SSC/bp	SSC percentage	IR/bp	IR percentage	Number of genes in the IR region	
									CDS	tRNA
*Ginkgo biloba*	156,988	39.6	99,254	63.2%	22,267	14.2%	17,732	11.3%	3	6
*Cycas revoluta*	162,489	39.4	88,978	54.8%	23,379	14.4%	25,066	15.4%	4	6
*Cycas panzhihuaensis*	162,470	39.4	88,932	54.7%	23,448	14.4%	25,045	15.4%	4	7
*Cycas szechuanensis*	162,083	39.4	88,970	54.9%	23,107	14.3%	25,003	15.4%	4	7
*Cycas taitungensis*	163,403	39.5	90,216	55.2%	23,039	14.1%	25,074	15.3%	4	7
*Bowenia serrulata*	165,695	39.9	90,733	54.8%	23,156	14.0%	25,903	15.6%	4	7
*Stangeria eriopus*	163,548	39.5	89,850	54.9%	23,006	14.1%	25,346	15.5%	4	7
*Ceratozamia hildae*	165,734	39.7	90,487	54.6%	22,973	13.9%	26,137	15.8%	4	7
*Dioon spinulosum*	161,815	40.1	88,754	54.8%	23,355	14.4%	24,853	15.4%	4	7
*Zamia furfuracea*	164,953	39.7	90,441	54.8%	23,228	14.1%	25,642	15.5%	4	7
*Encephalartos lehmannii*	165,822	39.9	90,724	54.7%	23,302	14.1%	25,898	15.6%	4	7
*Lepidozamia peroffskyana*	165,939	39.9	90,804	54.7%	23,299	14.0%	25,981	15.7%	4	7
*Macrozamia mountperriensis*	166,341	39.8	91,171	54.8%	23,334	14.0%	25,918	15.6%	4	7
*Gnetum montanum*	115,019	38.2	66,139	57.5%	9,494	8.3%	19,693	17.1%	3	8
*Gnetum parvifolium*	114,914	38.2	66,095	57.5%	9,559	8.3%	19,630	17.1%	4	8
*Gnetum ula*	113,249	38.5	64,914	57.3%	8,791	7.8%	19,772	17.5%	3	8
*Gnetum gnemon*	115,022	38.2	66,591	57.9%	8,329	7.2%	20,051	17.4%	5	8
*Ephedra equisetina*	109,518	36.6	59,936	54.7%	8,078	7.4%	20,752	18.9%	6	8
*Ephedra foeminea*	109,584	36.7	60,708	55.4%	8,078	7.4%	20,399	18.6%	6	8
*Ephedra intermedia*	109,667	36.6	59,936	54.7%	8,247	7.5%	20,742	18.9%	6	8
*Ephedra sinica*	109,550	36.7	59,961	54.7%	8,103	7.4%	20,743	18.9%	6	8
*Welwitschia mirabilis*	119,726	36.7	68,556	57.3%	11,156	9.3%	20,007	16.7%	5	8

Rank sum tests indicated no significant differences in genome size (*p* = .202) and significant differences in GC content (*p* < .01) between IR‐22 and SC‐56.

### 3.2 Phylogenetic analysis

The 50 protein‐coding genes comprised 30 photosynthetic system genes, 15 genetic systems, and 5 other genes (Table 3). The constructed phylogenetic results (Appendix S2) showed that the phylogenetic relationship constructed by the NJ method was the clearest, and each group formed a monophyletic branch. Using the BI and ML methods, we found that all groups, except the Pinales, formed a monophyletic branch. In the MP tree, the relationship between Cupressales and Araucariales was unclear.

**TABLE 3:** Types of common genes

Gene type	Gene name
Genes for photosynthesis	
Photosystem	*psaA psaB psaC psaI psaJ*
Photosystem II	*psbA psbB psbC psbD psbE psbF psbH psbI*
	*psbJ psbK psbL psbM psbN psbT*
Genetic system genes	
Cytochrome	*petA petB petD petG petL petN*
ATP Synthase	*atpB atpE atpF atpI*
RubiscoCO large subunit	*rbcL*
Ribosomal Proteins (LSU)	*rpl14 rpl20 rpl33 rpl36*
Ribosomal Proteins (SSU)	*rps2 rps4 rps7 rps8 rps11 rps18 rps19*
RNA Polymerase	*rpoA rpoB rpoC1 rpoC2*
Other genes	
Envelop membrane protein	*cemA*
C‐type cytochrome synthesis	*ccsA*
Hypothetical chloroplast reading frames	*ycf2 ycf3 ycf4*

**FIGURE 2:**
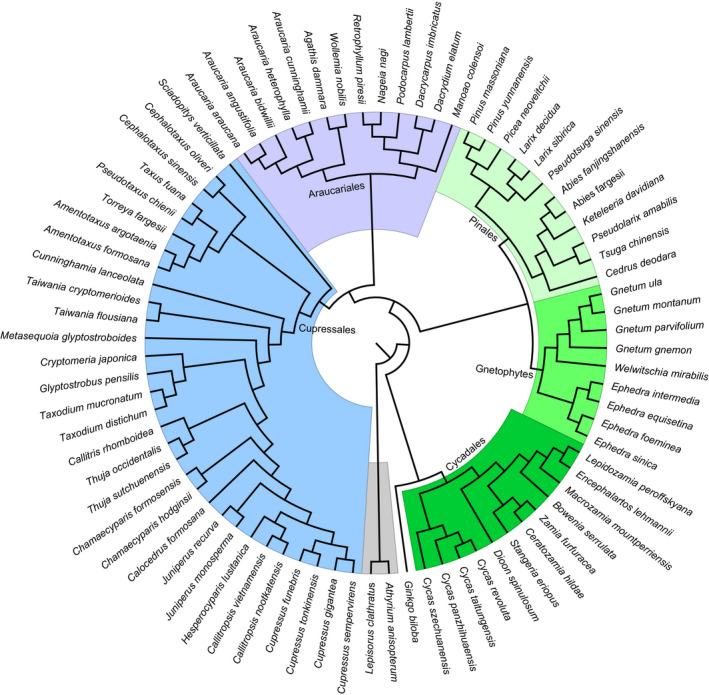
Phylogenetic relationship of sampled species. The tree was obtained through manual adjustment by combining four methods and previous research results. See Appendix S2 for four trees

The NJ (bootstrap value = 100) and MP (bootstrap value = 50) trees supported gnetophytes as the basic group of gymnosperms, and the MP (bootstrap value = 100) and BI (posterior probability = 1) trees supported gnetophytes and *P*. *neoveitchii* as sister groups. Based on previous reports, we accepted the *Gnepine* hypothesis, and performed a manual adjustment to obtain the phylogenetic tree for selection pressure and evolution rate analysis (Figure 2).

### 3.3 Evolutionary analysis based on the presence of a typical IR region

With IR‐22 as the foreground branch and SC‐56 as the background branch, likelihood ratio tests of M0 and Model 2 yielded 18 genes with significant differences (*p *< .05). That is, these 18 genes experience different selection pressure in IR‐22 and SC‐56. Among them, six genes had an *ω* value in IR‐22 that were lower than SC‐56, and the rest were higher (Table 4).

**TABLE 4:** Genes that accepted Model 2 in the Branch Model

Gene	*lnL* M0	*lnL* Model 2	2 Δ*Ɩ*	*P*‐value	*ω*_foreground_	*ω*_background_	*ω*_foreground/_ *ω*_background_
*atpF*	−6580.533	−6587.056	13.046	0	0.367	0.195	1.882
***ccsA***	−11747.691	−11749.697	4.012	.045	0.268	0.346	**0.775**
*cemA*	−10261.516	−10266.192	9.352	.002	0.418	0.272	1.537
***petD***	−3345.317	−3348.100	5.566	.018	0.066	0.117	**0.564**
*psaA*	−16296.555	−16299.080	5.050	.025	0.078	0.057	1.368
*psaJ*	−1520.374	−1522.340	3.932	.047	0.269	0.132	2.038
***psbA***	−6124.730	−6133.245	17.029	0	0.026	0.071	**0.366**
***psbC***	−9768.612	−9773.699	10.173	.001	0.042	0.073	**0.575**
***psbE***	−1676.534	−1681.768	10.468	.001	0.058	0.183	**0.317**
***psbJ***	−1229.013	−1231.721	5.416	.020	0.133	0.366	**0.363**
*psbM*	−1381.099	−1384.893	7.588	.006	0.479	0.132	3.629
*rpoB*	−38294.175	−38327.819	67.289	0	0.286	0.156	1.833
*rpoC1*	−26817.747	−26840.736	45.978	0	0.357	0.191	1.869
*rpoC2*	−50272.622	−50279.519	13.795	0	0.372	0.294	1.265
*rps2*	−8506.349	−8508.885	5.071	.024	0.281	0.205	1.371
*rps11*	−4972.071	−4979.642	15.142	0	0.339	0.152	2.230
*rps18*	−5208.180	−5212.761	9.163	.002	0.352	0.160	2.200
*ycf2*	−142683.719	−142688.968	10.499	.001	0.768	0.635	1.209

Note

Foreground is IR‐22 and background is SC‐56; Bold font indicates that *ω*_foreground_ is less than *ω*_background_.

**FIGURE 3:**
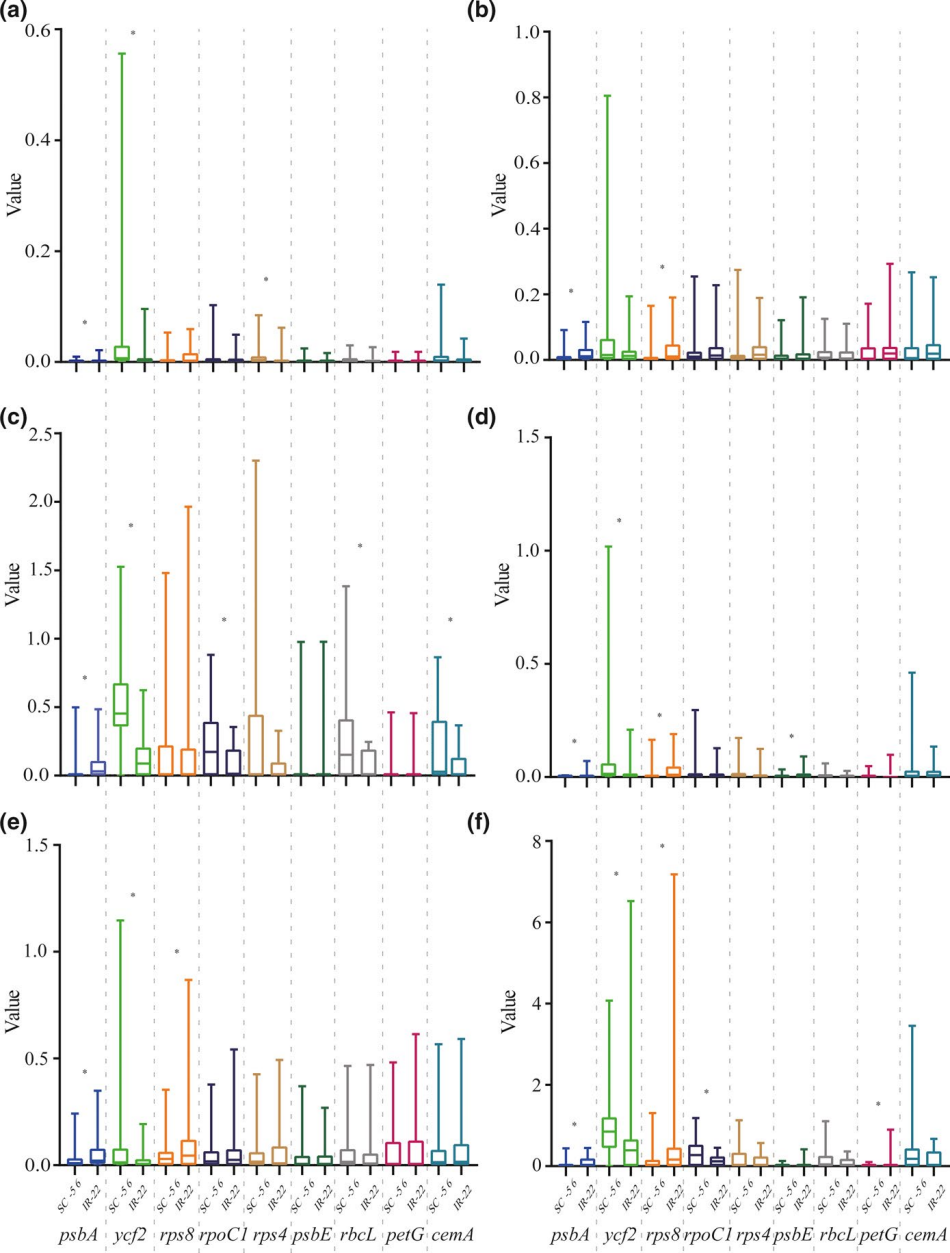
Nine of fifty common genes with significantly different evolutionary rates between IR‐22 (non‐conifers) and SC‐56 (conifers). (a) Transversion rate. (b) Transition rate. (c) Ratio. (d) Non‐synonymous substitution rate. (e) Synonymous substitution rate. (f) *ω*. *: Rank sum test, *p* < .05. Compared with SC‐56, the genes with a higher evolution rate in IR‐22 were *trst*, *trsv*, *ratio*, *dN*, *dS*, and *ω* of *psbA*; *trst*, *dN*, *dS*, and *ω* of *rps8*; *dN* of *psbE*; and *ω* of *petG*. Compared with IR‐22, the genes with higher evolution rates in the SC‐56 were *trsv*, *ratio*, *dN*, *dS*, and *ω* of *ycf2*; *trsv* of *rps4*; and ratios of *rpoC1*, *rbcL*, and *cemA*

We analyzed and tested the evolutionary rates of the 50 selected common genes (Appendix S3). Among these, we found that the related parameters of nine genes were significantly different between IR‐22 and SC‐56 (*p* < .05; Figure 3). Compared with SC‐56, the genes with a higher evolution rates in IR‐22 were *trst* (.003 vs. .001), *trsv* (.026 vs. .010), *ratio* (.082 vs. .031), d*N* (.007 vs. .001), d*S* (.070 vs. .025), and *ω* (.100 vs. .032) of *psbA* (2.6‐ to 4.2‐fold that of the SC‐56); *trst* (.052 vs. .021), d*N* (.033 vs. .014), d*S* (.112 vs. .051), and *ω* (.631 vs. .226) of *rps8* (2.3‐ to 2.4‐fold); d*N* (.010 vs. .002) of *psbE* (5‐fold); and *ω* (.064 vs. .002) of *petG* (32‐fold). Compared with IR‐22, the genes with higher evolution rates in the SC‐56 were *trsv* (.033 vs. .009), *ratio* (.504 vs. .145), d*N* (.063 vs. .022), d*S* (.068 vs. .024), and *ω* (.904 vs. .024) of *ycf2* (1.2‐ to 3.6‐fold those of IR‐22); *trsv* (.008 vs. .005) of *rps4* (1.8‐fold); and ratios of *rpoC1* (.224 vs. .087), *rbcL* (.234 vs. .077), and *cemA* (.183 vs. .064) (2.6‐, 3‐, and 2.8‐fold, respectively). Spearman's rank correlation test results showed that the evolutionary rate of genes other than *dS* of *rps8* (Spearman's rho = .157, *p* = .169) and *ω* of *psbA* (Spearman's rho = .212, *p* = .063) was significantly correlated (*p* < .05) with the IR region (Appendix S4).

The evolutionary rate of *psbA* (Appendix S5) was abnormally high in *Cycas taitungensis* and *G*. *biloba* and in *Cycas* spp. By contrast, the species evolutionary rate of SC‐56 was low. For *rps8* (Appendix S6), evolutionary rates were high in *G*. *biloba*, *W*.* mirabilis*, and cycads; the values for most other species branches in SC‐56 were 0, except *C*. *rhomboidea* and *S*. *verticillata*. For *rps4* (Appendix S7A), the transversion rate of IR‐22 was low, and most values were 0. All species branches in SC‐56 had high transversion rates, with those of *C*. *rhomboidea* and *S*. *verticillata* being abnormally high. For *psbE* (Appendix S7B), the d*N* of IR‐22 was high (especially in *W*. *mirabilis* and *Dioon spinulosum*) and was 0 for most species in SC‐56 (*n* = 49). For *ycf2* (Appendix S8), substitution rates were high in most branches of SC‐56, especially *C*. *rhomboidea* and *S*.* verticillata*. In IR‐22, the value of the other branches was 0, except in *W*. *mirabilis* and *G*. *biloba*.

### 3.4 Evolutionary analysis based on genes entering the IR region

The IR regions of 22 species contained the protein‐coding genes 3’‐*rps12* and *rps7* (Figure 1). In *W*. *mirabilis*, *G*. *parvifolium*, and *G*. *gnemon*, part of the *psbA* (3’‐*psbA*) entered the IR region; thus, it was divided into two categories (IR‐3 and SC‐75). In *G*. *biloba*, *ycf2* has been left from the IR region; thus, it was divided into two categories (IR‐21 and SC‐57).

**FIGURE 4:**
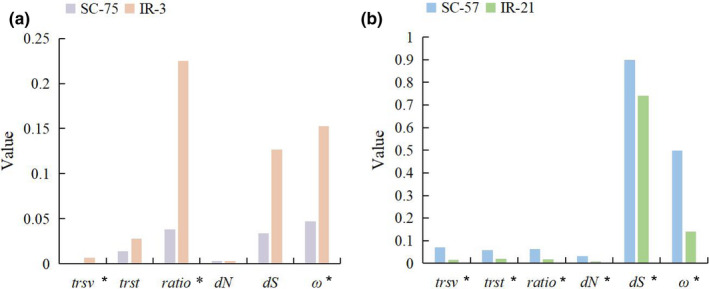
The evolutionary rate of *psbA* and *ycf2* in the different categories. (a) For *psbA*, the mean evolutionary rate of IR‐3 and SC‐75. B: For *ycf2*, the mean evolutionary rate of IR‐21 and SC‐57. *Rank sum test, *p* < .05. The *trsv*, *ratio*, and *ω* values of *psbA* were significantly higher in the IR region than in the SC region, and the evolutionary rate of *ycf2* was significantly lower in the IR region than in the SC region

Likelihood ratio test results revealed that the selection pressure differed among categories. For *psbA*, *ω*_IR‐3_ was 0.03474, with IR‐3 as the foreground branch, and *ω*_SC‐75_ was 0.0001, with SC‐75 as the background branch. For *ycf2*, *ω*_IR‐21_ was 0.76124, with IR‐21 as the foreground branch, and *ω*_SC‐57_ was 0.67832, with SC‐57 as the background branch. The *trsv*, *ratio*, and *ω* values of *psbA* were significantly higher (*P_trsv_* = .048, *P_ratio_* = .003, and *P_ω_* = .027) in the IR region than in the SC region, and the evolutionary rate of *ycf2* was significantly lower (*p* < .05) in the IR region than in the SC region (Figure 4).

## 4 DISCUSSIONS

### 4.1 Changes in genome size

Evolution has decreased the gymnosperm genome size. Conifers and non‐conifers have genomes of similar sizes, which is related to the large compact genome of gnetophytes (115,022–109,518 bp). This compact genome may increase the gnetophyte survival rate in harsh and competitive environments (Wu et al., 2009). After the removal of gnetophytes, a significant difference was detected between groups (*p* < .01). The shrinkage of the conifer plastome is a result mainly of the loss of the IR region (IRb in Pinaceae and IRa in cupressophytes). In addition, the reduction in the cupressophyte plastome size may be caused by intergenic shrinkage resulting from the mutational burden (Wu & Chaw, 2014). Pinaceae and cupressophytes have lost their typical IRs during evolution, but have acquired one or more short, novel IRs, some of which also exhibit recombination to generate genomic structural diversity (Guo et al., 2014).

Compared with that of ginkgo, the IR region of other non‐conifers has expanded. Although the IR region in gnetophytes is smaller than that in cycads, it accounts for a large proportion of the genome and contains more genes. Gnetophytes lost the *ndh* gene and non‐coding sequence, resulting in a compact, more economical genome (Wu et al., 2009). In Pinaceae, the IR comprises only the full *trnI*‐*CAU* gene and, in most species, a portion of the *psbA* gene (Lin et al., 2010; Wu, Lin, et al., 2011).

The GC content was increased in non‐conifers, which may be related to a GC bias in gene conversion in the IR region. Indeed, the IR region of cycads contains more A/T to GC substitutions (Wu & Chaw, 2015).

### 4.2 Phylogenetic location of gnetophytes

The four phylogenetic trees constructed using a tandem dataset of 50 common protein‐coding genes were not consistent, and the application of different algorithms yielded different topological structures. Compared with those produced by NJ and MP, the phylogenetic relationships constructed by ML and BI are more accurate (Hall, 2005). The ML and BI trees showed that gnetophytes and *P*. *neoveitchi* were sister groups with a high degree of support. Although Pinaceae was a non‐monophyletic group, it was closest to the gnetophytes, supporting the *Gnepine* hypothesis.

The phylogenetic position of the gnetophytes has been debated (Mathews, 2009; Palmer et al., 2004). Burleigh and Mathews (2004) examined 13 genes in gymnosperms (five plastid, four nuclear, and four mitochondrial) and almost 19 000 nucleotides, providing evidence for the *Gnepine* hypothesis. Wu and Chaw (2014) analyzed the rearrangement of DNA in the plastome of gymnosperms, and the results supported the *Gnepine* hypothesis. Li et al. (2017) analyzed single‐copy genes from the genome and transcriptome, and the phylogenetic relationships constructed from their different datasets supported two topological structures: a sister relationship between gnetophytes and other gymnosperms and gnetophytes as a sister group to Pinaceae. Ran et al. (2018) constructed a phylogenetic tree with 1308 loci based on transcriptome data, which also supported the *Gnepine* hypothesis. Ping, Feng, et al. (2021) constructed ML and BI trees using *rbcL* and *matK*, which supported the classification of gnetophytes and Pinaceae as sister groups.

### 4.3 Heterogeneity of evolutionary rates

The change in evolutionary rate was independent of the presence of the IR region, but rather indicates an increased substitution rate of gnetophytes (especially *W*. *mirabilis*) and a lower evolutionary rate of conifers. The evolutionary rate of four genes was increased in non‐conifers. The increased substitution rates of *rps8*, *psbE*, and *psbA* were related to the low evolutionary rate of conifers. The *petG* value was abnormally high. The *dN* or *dS* value for most species branches was 0, resulting in false‐positive results. By contrast, the substitution rates of *rps4* and *ycf2* were decreased in non‐conifers, which is related to their generally low evolutionary rate. The evolutionary rate of most genes in *W*. *mirabilis* was high. The phylogenetic tree constructed using 51 common genes showed that the gnetophytes, especially *W*. *mirabilis* and *Ephedra sinica*, had high evolutionary rates. McCoy et al. (2008) reported that about 75% of plastid protein‐coding genes showed high substitution rates in *W*. *mirabilis*. The substitution rates of most protein‐coding genes did not differ significantly between conifers and non‐conifers, which may be related to the newly acquired short IR region of conifers (Hirao et al., 2008; Wu & Chaw, 2014; Yi et al., 2013). In *Cephalotaxus oliveri*, the 544‐bp IR (repeated *trnQ*‐*UUG* gene) was inferred to have recombination activity (Yi et al., 2013).

Gnetophytes have a higher substitution rate than do other gymnosperms (Ran et al., 2018; Wang et al., 2015; Wu et al., 2009), possibly because of higher mutation rates, changes in selective pressure, increased fixation of mutations by genetic drift, and biological characteristics (Fry & Wernegreen, 2005; Lanfear et al., 2010). Wu et al. (2009) proposed that the high frequency of AT‐rich codons in gnetophytes leads to a higher substitution rate. Wang et al. (2015) proposed that the generation time and plant height underlie the increased substitution rate of gnetophytes (Lanfear et al., 2010, 2013). Ran et al. (2018) found that gnetophytes and angiosperms have similar rates of molecular evolution, which are higher than those of other gymnosperms, suggesting that gnetophytes and angiosperms experienced similar selection pressure during their evolutionary histories. As conifers are typically taller than non‐conifers, the long‐term rate of mitosis in the apical meristem is slower, the frequency of DNA replication is lower, and the accumulated errors per unit time are reduced, resulting in low mutation and substitution rates (Lanfear et al., 2013).

### 4.4 Negative selection pressure

Genes in the IR region experience strong negative selection (Ping, Feng, et al., 2021; Ping, Li, et al., 2021). The IR region is important for genome structural stability, and genomes lacking an IR region may experience different natural selection effects. However, the selection pressure of most genes did not differ significantly, indicating that they are not affected by the IR region. When *ω* < 1, smaller *ω* values reflect stronger negative selection pressure (Yang, 2006). The selection pressure of 18 genes differed between conifers and non‐conifers, and their *ω* values were <1 (Table 4). Compared with conifers, six genes (four PSII genes, one cytochrome gene, and one c‐type cytochrome synthesis gene) showed lower *ω* values in non‐conifers, and 12 genes (three PSI genes, one PSII gene, six genetic system genes, and two other genes) showed higher *ω* values in non‐conifers. The lower *ω* values of these genes indicates the greater the negative selection effect, which may be more conducive to their function in various groups. The differences in selection pressure between conifers and non‐conifers may be related to plant height; non‐conifers are typically shorter than conifers. Efficient photosynthesis is required to obtain enough sunlight, necessitating negative selection pressure on the genes encoding the photosynthetic machinery. However, some such genes in conifers experienced negative selection pressure, which may be related to their structure or function or to habitat differences. The negative selection exerted by the habitats of gnetophytes leads to gene‐specific reductions in *dN*/*dS* in their plastomes and genomes (Wang et al., 2015).

### 4.5 Reduced substitution rate in the IR region

Genes in the IR region tend to have a low substitution rate (Li, Kuo, et al., 2016; Li, Gao, et al., 2016; Ping, Feng, et al., 2021; Ping, Li, et al., 2021). In this study, *ycf2* in the IR region showed a reduced substitution rate (Figure 4) but weak selection pressure (*ω*_IR‐21_ = 0.76124, *ω*_SC‐57_ = 0.67832), indicating the lack of correlation between these factors. However, Lin et al. (2012) showed that the substitution rate of *ycf2* in the SC region of *G*. *biloba* was not significantly higher than that in the IR region in seven other species. These discrepancies in results are attributable to differences in sample size. In gymnosperms, 3'‐*rps12* (exons 2–3) shows a reduced substitution rate and negative selection pressure when present in the IR region (Ping, Feng, et al., 2021).

The substitution rate and selection pressure of *rps7* in the IR region did not change, indicating high stability during gymnosperm evolution. The other gene, *psbA*, encodes the D1 protein in the PSII core complex. In ferns, *psbA*, *ycf2*, *rps7*, and 3'‐*rps12* (exons 2–3) showed reduced substitution rates when present in the IR region (Li, Kuo, et al., 2016; Li, Gao, et al., 2016). *psbA* only partially enters the IR region in three gymnosperm species and shows increased substitution rates (Figure 4) and weak selection pressure. These characteristics differ from those of other genes; the most reasonable explanation is that this gene is undergoing dynamic changes in gymnosperms, resulting in changes in its substitution rate and selection pressure.

IR regions may play an important role in the maintenance of genomic stability (Maréchal & Brisson, 2010), such as by replication initiation (Heinhorst & Cannon, 1993), genome stabilization (Palmer & Thompson, 1982), and gene conservation (Palmer & Thompson, 1982; Wolfe et al., 1987). IR loss is related to structural recombination. In some taxa lacking an IR region, plastids underwent significant structural changes, including gene and intron loss, multiple inversion, and translocation and duplication of plastome segments (Jin et al., 2020; Hirao et al., 2008; Mower & Vickrey, 2018). Sequences in the IR region often have a reduced substitution rate, which is related to the region's characteristics. Due to its double‐copy nature, the frequency of gene conversion is higher, and some mutation sites are repaired, resulting in a low substitution rate (Birky & Walsh, 1992; Khakhlova & Bock, 2006).

## CONFLICT OF INTEREST

The authors declare no conflict of interest.

## AUTHOR CONTRIBUTION

**Jingyao Ping:** Conceptualization (equal); Data curation (equal); Formal analysis (equal); Investigation (equal); Methodology (equal); Writing – original draft (equal). **Jing Hao:** Data curation (equal); Formal analysis (equal); Methodology (equal). **Jinye Li:** Data curation (equal); Formal analysis (equal); Resources (equal). **Yiqing Yang:** Formal analysis (equal); Methodology (equal). **Yingjuan Su:** Funding acquisition (equal); Writing – original draft (equal); Writing – review & editing (equal). **Ting Wang:** Funding acquisition (equal); Writing – original draft (equal); Writing – review & editing (equal).

## Supporting information

Appendix S1

Appendix S2

Appendix S3

Appendix S4

Appendix S5

Appendix S6

Appendix S7

Appendix S8

## Data Availability

Data source is NCBI database: https://www.ncbi.nlm.nih.gov/nuccore/NC_020319 https://www.ncbi.nlm.nih.gov/nuccore/NC_031413 https://www.ncbi.nlm.nih.gov/nuccore/NC_042668 https://www.ncbi.nlm.nih.gov/nuccore/NC_009618 https://www.ncbi.nlm.nih.gov/nuccore/NC_026036 https://www.ncbi.nlm.nih.gov/nuccore/NC_026041 https://www.ncbi.nlm.nih.gov/nuccore/NC_026037 https://www.ncbi.nlm.nih.gov/nuccore/NC_027512 https://www.ncbi.nlm.nih.gov/nuccore/NC_026040 https://www.ncbi.nlm.nih.gov/nuccore/NC_027514 https://www.ncbi.nlm.nih.gov/nuccore/NC_027513 https://www.ncbi.nlm.nih.gov/nuccore/NC_027511 https://www.ncbi.nlm.nih.gov/nuccore/NC_016986 https://www.ncbi.nlm.nih.gov/nuccore/NC_021438 https://www.ncbi.nlm.nih.gov/nuccore/NC_011942 https://www.ncbi.nlm.nih.gov/nuccore/NC_028734 https://www.ncbi.nlm.nih.gov/nuccore/NC_026301 https://www.ncbi.nlm.nih.gov/nuccore/NC_011954 https://www.ncbi.nlm.nih.gov/nuccore/NC_029347 https://www.ncbi.nlm.nih.gov/nuccore/NC_044772 https://www.ncbi.nlm.nih.gov/nuccore/NC_044773 https://www.ncbi.nlm.nih.gov/nuccore/EU342371 https://www.ncbi.nlm.nih.gov/nuccore/NC_010548 https://www.ncbi.nlm.nih.gov/nuccore/NC_016065 https://www.ncbi.nlm.nih.gov/nuccore/NC_021441 https://www.ncbi.nlm.nih.gov/nuccore/NC_021437 https://www.ncbi.nlm.nih.gov/nuccore/NC_024022 https://www.ncbi.nlm.nih.gov/nuccore/NC_042763 https://www.ncbi.nlm.nih.gov/nuccore/NC_034941 https://www.ncbi.nlm.nih.gov/nuccore/NC_045277 https://www.ncbi.nlm.nih.gov/nuccore/NC_023121 https://www.ncbi.nlm.nih.gov/nuccore/NC_039562 https://www.ncbi.nlm.nih.gov/nuccore/NC_028155 https://www.ncbi.nlm.nih.gov/nuccore/NC_026296 https://www.ncbi.nlm.nih.gov/nuccore/MT227813 https://www.ncbi.nlm.nih.gov/nuccore/KP099642 https://www.ncbi.nlm.nih.gov/nuccore/KX832629 https://www.ncbi.nlm.nih.gov/nuccore/MH121051 https://www.ncbi.nlm.nih.gov/nuccore/NC_034943 https://www.ncbi.nlm.nih.gov/nuccore/NC_036996 https://www.ncbi.nlm.nih.gov/nuccore/NC_031354 https://www.ncbi.nlm.nih.gov/nuccore/NC_027423 https://www.ncbi.nlm.nih.gov/nuccore/NC_042176 https://www.ncbi.nlm.nih.gov/nuccore/NC_042177 https://www.ncbi.nlm.nih.gov/nuccore/NC_034940 https://www.ncbi.nlm.nih.gov/nuccore/MF977938 https://www.ncbi.nlm.nih.gov/nuccore/NC_021110 https://www.ncbi.nlm.nih.gov/nuccore/NC_027581 https://www.ncbi.nlm.nih.gov/nuccore/NC_024945 https://www.ncbi.nlm.nih.gov/nuccore/NC_038099 https://www.ncbi.nlm.nih.gov/nuccore/NC_041503 https://www.ncbi.nlm.nih.gov/nuccore/NC_029398 https://www.ncbi.nlm.nih.gov/nuccore/NC_029734 https://www.ncbi.nlm.nih.gov/nuccore/NC_014575 https://www.ncbi.nlm.nih.gov/nuccore/MF564195 https://www.ncbi.nlm.nih.gov/nuccore/NC_043856 https://www.ncbi.nlm.nih.gov/nuccore/NC_030631 https://www.ncbi.nlm.nih.gov/nuccore/NC_016064 https://www.ncbi.nlm.nih.gov/nuccore/NC_043913 https://www.ncbi.nlm.nih.gov/nuccore/NC_011930 https://www.ncbi.nlm.nih.gov/nuccore/NC_030630 https://www.ncbi.nlm.nih.gov/nuccore/NC_036811 https://www.ncbi.nlm.nih.gov/nuccore/NC_016058 https://www.ncbi.nlm.nih.gov/nuccore/NC_042775 https://www.ncbi.nlm.nih.gov/nuccore/NC_042777 https://www.ncbi.nlm.nih.gov/nuccore/NC_023120 https://www.ncbi.nlm.nih.gov/nuccore/NC_023805 https://www.ncbi.nlm.nih.gov/nuccore/NC_034942 https://www.ncbi.nlm.nih.gov/nuccore/NC_024827 https://www.ncbi.nlm.nih.gov/nuccore/NC_045880 https://www.ncbi.nlm.nih.gov/nuccore/NC_044893 https://www.ncbi.nlm.nih.gov/nuccore/NC_023119 https://www.ncbi.nlm.nih.gov/nuccore/KP259800 https://www.ncbi.nlm.nih.gov/nuccore/MT227812 https://www.ncbi.nlm.nih.gov/nuccore/NC_039155 https://www.ncbi.nlm.nih.gov/nuccore/NC_026450 https://www.ncbi.nlm.nih.gov/nuccore/NC_045394 https://www.ncbi.nlm.nih.gov/nuccore/NC_045395 https://www.ncbi.nlm.nih.gov/nuccore/NC_035739 https://www.ncbi.nlm.nih.gov/nuccore/NC_035738
