# Supplementary figures and images for: Loss of the IR region in conifer plastomes: Changes in the selection pressure and substitution rate of protein‐coding genes
Source: Ecol Evol. 2022 Jan 12;12(1):e8499. doi: 10.1002/ece3.8499 (PMC8809450; doi:10.1002/ece3.8499)

Appendix S2 Phylogenetic tree based on shared genes. A-D: NJ, MP, ML, BI.


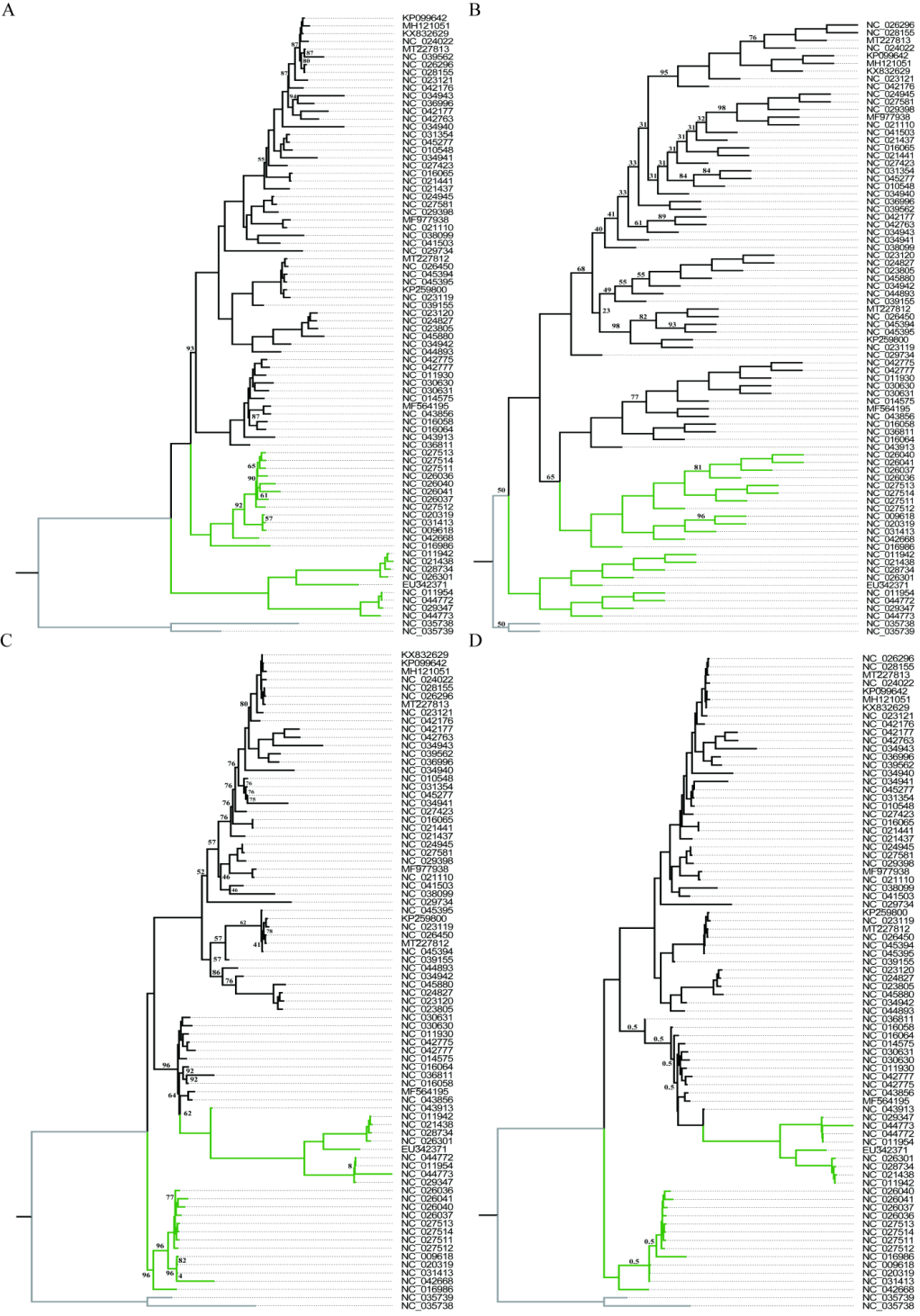

Supplement: Supplementary file 2 — Appendix S2 [file ECE3-12-e8499-s003.doc]

Appendix S5 Evolutionary rate tree of *psbA* A-D: transversion, transition, *dN*, *dS.*


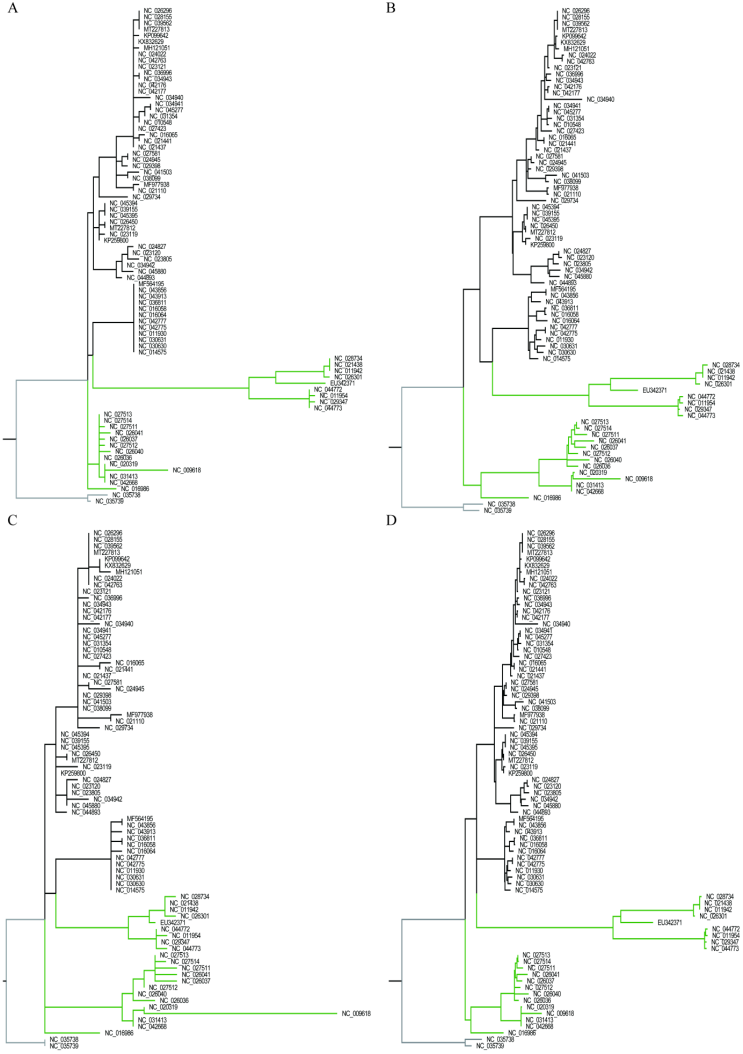

Supplement: Supplementary file 5 — Appendix S5 [file ECE3-12-e8499-s001.doc]

Appendix S6 Evolutionary rate tree of *rps8*.A-C: transition, *dN*, *dS.*


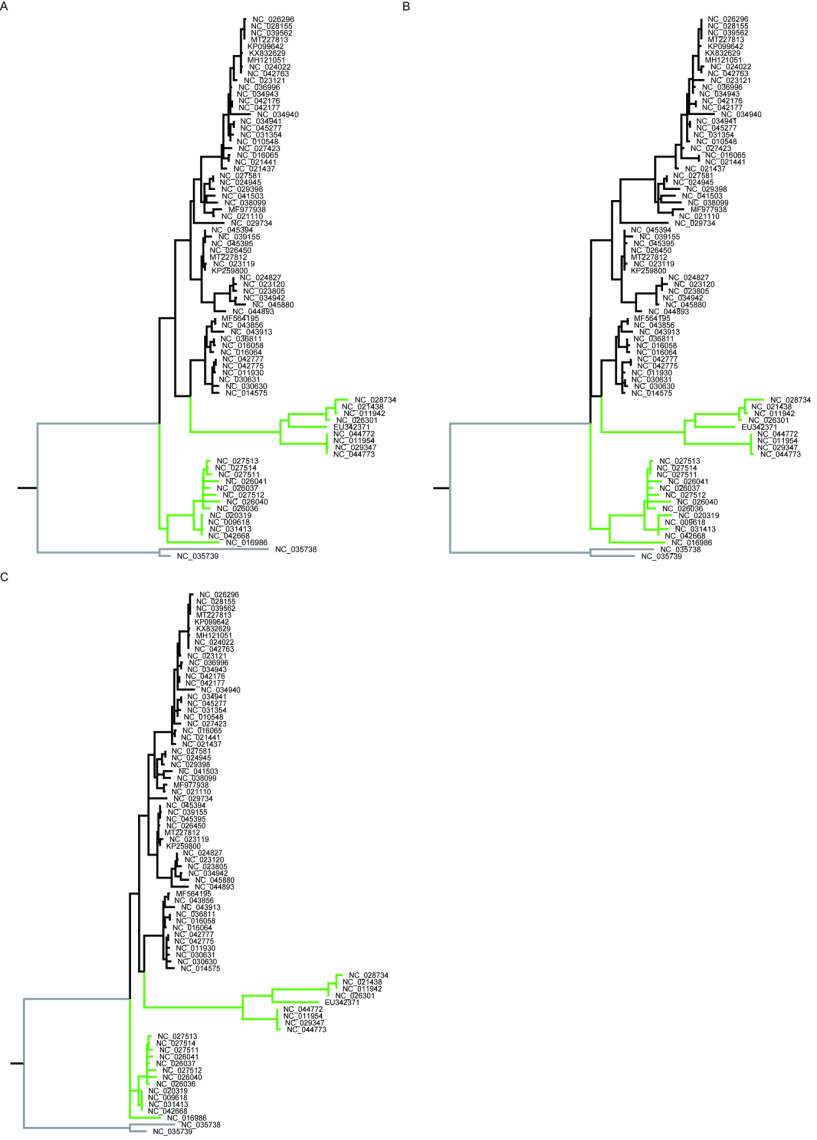

Supplement: Supplementary file 6 — Appendix S6 [file ECE3-12-e8499-s002.doc]

Appendix S8 Evolutionary rate tree of *ycf2* A-D: transversion, transition, *dN*, *dS.*


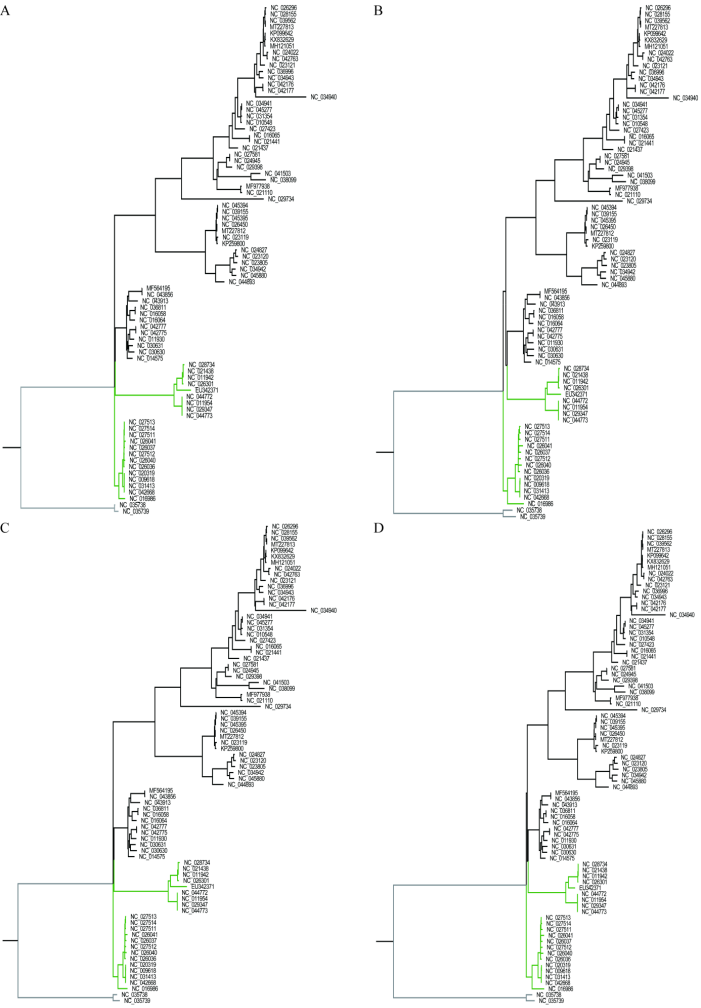

Supplement: Supplementary file 8 — Appendix S8 [file ECE3-12-e8499-s006.doc]
