# Supplementary material for: Loss of the IR region in conifer plastomes: Changes in the selection pressure and substitution rate of protein‐coding genes
Source: Ecol Evol. 2022 Jan 12;12(1):e8499. doi: 10.1002/ece3.8499 (PMC8809450; doi:10.1002/ece3.8499)

Appendix S7 Evolutionary rate tree of *rps4* and *psbE* A: the transversion tree of *rps4*; B: the *dN* tree of *psbE*.


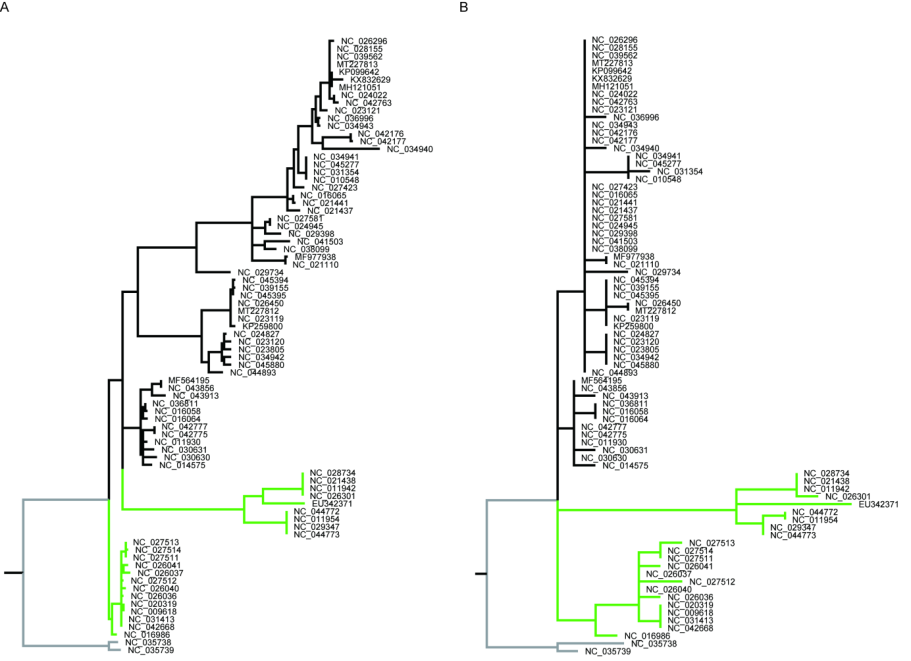

Supplement: Supplementary file 7 — Appendix S7 [file ECE3-12-e8499-s005.doc]
